# Boredom and its perceived impact in adolescents with exceptional mathematical talent: a sequential mixed-methods study in Paraguay

**DOI:** 10.3389/fsoc.2024.1214878

**Published:** 2024-05-24

**Authors:** Alexandra Vuyk, Maureen Montania, Liz Barrios

**Affiliations:** ^1^Aikumby Centro de Altas Capacidades y Creatividad, Asunción, Paraguay; ^2^OMAPA – Organización Multidisciplinaria de Apoyo a Profesores y Alumnos, Asunción, Paraguay; ^3^Faculty of Philosophy and Human Sciences, Department of Psychology, Asunción, Paraguay; ^4^Faculty of Economical Sciences, School of Economics, Asunción, Paraguay

**Keywords:** boredom, mathematical talent, talented adolescents, Paraguay, mixed methods

## Abstract

**Introduction:**

Boredom, a state where the task at hand presents difficulties in attentional resources and attributed meaning, can be detrimental to talent development by reducing cognitive engagement. This study employed a mixed sequential design to assess boredom in adolescents with exceptional mathematical talent in Paraguay participating in a talent development program.

**Methods:**

First, in the quantitative phase 54 students completed the Boredom Short Scale, School Attitudes Assessment Survey-Revised, and Psychological Well-Being Scale for Adolescents. Next, in the qualitative phase 50 students participated in focus groups to explore their personal experiences of boredom, along with their perception of possible factors that impact boredom.

**Results:**

Boredom in this population was similar to the general population of adolescents, albeit significant differences existed in items and subscales showing a pattern unique to this population; they tended to become bored quicker but had an easier time sparking interest in activities. Higher boredom had small to moderate correlations with worse attitudes at school and with teachers, lower motivation and self-regulation in academic activities, lower self-control, and lower involvement in personal projects. Higher valuation of goals and academic self-perception related with a lower tendency toward boredom only in the context of a talent development program, but not at school. Boredom seemed multifaceted, with dimensions such as the absence of meaning, superficial entertainment, and wasted time. Contributing factors included waiting for other people’s slower pace, limited choices, lack of novelty, insufficient intellectual challenge, and the influence of teachers and social dynamics. Coping mechanisms included inner intellectual stimulation, seeking entertainment and escape, pursuing independent learning, extracurricular activities, like-minded peers, and extreme sensation-seeking.

**Discussion:**

Fostering adequate challenge and support in advanced academic endeavors for the development of mathematical talent can prevent negative consequences associated with boredom in exceptionally talented populations.

## Introduction

Boredom is a subjective experience that arises when an individual feels disengaged or uninterested in the task at hand. Being bored means that we want to do something else in that moment; this negative emotional state serves as a sign of a longing to pursue alternative activities (Westgate and Wilson, 2018). Boredom can lead to disengagement from schoolwork among talented students. In the case of mathematically talented students, insufficiently challenging classroom environments can trigger them to become heavily bored and detached from schoolwork; in turn, this could lead to decreased motivation, poor academic performance, and behavior problems that might prevent them from engaging with their communities (Wegner and Flisher, 2009; Elia et al., 2009; Leikin, 2011; Singer et al., 2016; Kilic et al., 2019; Leikin, 2020). Long-term effects might include limited development of critical thinking skills and creativity, both of which are necessary for success in any domain. Hence, it is critical to tackle boredom and ensure that mathematically talented students are appropriately challenged and supported in their academic endeavors (Smedsrud et al., 2022).

### Boredom in the Meaning and Attentional Components Model

The Meaning and Attentional Components (MAC) model explains boredom in the general population as it occurs while engaged in an external activity or in one’s own thoughts. This model has the advantage of integrating prior approaches that focus separately on deficits in meaning or attention. The MAC model of boredom offers a broader perspective compared to previous theories by encompassing a wider range of experiences and outcomes associated with boredom. It not only provides an explanation for boredom but also generates new predictions by recognizing different types of boredom that can elicit distinct responses based on the underlying causes related to meaning and attention. This expanded view allows for a more comprehensive understanding of the complexities of boredom and its implications (Westgate and Wilson, 2018).

The MAC model suggests that mixed states of boredom occur when individuals experience deficits in both attention and meaning. These states are commonly observed in tasks that lack significance and fail to provide adequate stimulation. Attention and meaning are recognized as separate causes of boredom, and when attentional fit is achieved, individuals engage cognitively when their cognitive demands are appropriately met (Zárate, 2021). This cognitive engagement can manifest in two ways: low-level engagement and high-level engagement, which correspond to situations of understimulation and overstimulation, respectively. The MAC model highlights that understimulation, characterized by either low demands or high resources, strongly predicts boredom. This lack of challenge, where resources surpass demands, is identified as a key factor in boredom in both recent studies (Van Tilburg and Igou, 2017) and established theoretical models (Csikszentmihalyi, 2000).

#### Alleviating boredom

Strategies against boredom aim to choose interesting activities over enjoyable ones to alleviate boredom and promote long-term wellbeing. The MAC model suggests four primary routes to alleviating boredom. These include regulating cognitive demands, regulating cognitive resources, regulating value of the goal, and switching activities. Regulating cognitive demands requires making the task difficult enough until it is a good fit to reduce attentional boredom; plus, it can involve adding external attentional demands, such as listening to the radio, snacking, creative mind-wandering, or other similar tasks (Wilson et al., 2014; Westgate, 2020).

In the short-term, individuals can regulate their cognitive resources by employing physiological measures such as consuming caffeine or getting adequate sleep. These options help enhance attention and alleviate boredom. On the other hand, long-term strategies for regulating cognitive resources involve engaging in sustained practice and skill development. By consistently practicing and developing skills, individuals can effectively manage their cognitive resources and reduce the likelihood of experiencing boredom over extended periods of time (Walsh et al., 1993).

The regulation of goal values involves mentally reinterpreting activities to fill them with greater meaning and introducing new goals that counteract the potential monotony associated with repetitive tasks (Wilson, 2011). This process entails reframing one’s perspective and finding ways to assign significance and purpose to the activities at hand. By actively adjusting the perceived value and introducing fresh objectives, individuals can mitigate the risk of monotony and enhance their engagement and interest in the tasks they are undertaking (Steinberger et al., 2017). Engaging in different activities can be an effective strategy for managing boredom as well; however, this activity is not always possible.

Boredom levels of reward sensitivity and impulsivity can be diverted to unhealthy paths when self-regulation skills are not practiced (Moynihan et al., 2017; Milyavskaya et al., 2019), making low-effort enjoyable alternatives such as mood-altering drugs (e.g., alcohol, marijuana, tobacco) more appealing (Weybright et al., 2015). This is where choosing interesting activities over enjoyable ones becomes critical, as the former promote short-term happiness at the expense of long-term well-being. For example, choosing a fun and easy activity (like watching short videos on social media) over a more difficult but intriguing one (like watching a documentary film) might mitigate feelings of boredom in the moment, but does not enhance the cognitive work needed to develop self-regulation skills and prevent boredom in similar situations in the future.

In this model, interest and enjoyment are separate experiences. To increase interest, introducing elements of novelty and complexity can be beneficial. On the other hand, when seeking enjoyment, it is advantageous to opt for activities that provide a sense of certainty. Therefore, the choice of activities should be based on how one desires to feel, whether it is a desire for heightened interest or enhanced enjoyment (Silvia, 2006).

### Boredom in talented students

In the field of gifted education, there is no universally accepted definition of giftedness or talent, leading to a diverse array of terminologies and conceptualizations across different countries and states (Peters et al., 2014). Giftedness always encompasses exceptional talent, yet the specific terms used to describe these individuals can vary depending on the educational paradigm and cultural context (Dai and Chen, 2014). In this study, we follow the talent development paradigm (Dai and Chen, 2014) and use the terms ‘gifted’ and ‘talented’ to refer broadly to students who demonstrate advanced academic abilities and potential, recognizing that these labels may carry different connotations in different settings. Additionally, we chose to maintain the terminology used in the cited studies for consistency with the original sources.

Talent development programs recognize the importance of motivation and interest and seek to cultivate and nurture these aspects. Talent development considers abilities as evolving over time (Renzulli et al., 2009; Subotnik et al., 2011). Initially, talent is seen as the potential for future accomplishments, often observed in young children. As children grow and progress into adolescence, this potential transforms into competence and expertise. The final stages of talent development involve creative productivity and/or eminence, typically realized in adulthood (Subotnik et al., 2011). The educational journey of students can vary depending on their early environment and exposure to subjects like math and science. Some students, especially those who experience high levels of boredom, may possess exceptional learning potential that is not necessarily reflected in advanced knowledge or achievements. Studies by Dai (2010), Farrington et al. (2012), and Baudson and Preckel (2013) provide insights into this phenomenon.

Special programs are needed to address boredom and fully develop talents (Olszewski-Kubilius et al., 2014). When gifted and talented students feel bored or underchallenged, they rarely ask their teachers for help, favoring behavioral-avoidance coping mechanisms instead (Hinterplattner et al., 2022). Boredom represents a cross-sectional factor that contributes to underachievement in the regular curriculum in elementary and middle school (Preckel et al., 2010).

Neuroscience studies with mathematically gifted adolescents evidenced that the complexity of the task is related to brain activation and efficient processing (Zhang et al., 2017). In academic settings, inefficient processing due to low or high arousal translates into task-focus boredom and/or self-focus boredom. Task-focused boredom is characterized by an emphasis on the tediousness and meaninglessness of the task, whereas self-focused boredom is regarded by feelings of dissatisfaction and/or frustration (Smedsrud et al., 2022). Students who are prone to boredom may be more aware of their own internal state and may be better at recognizing when they are feeling bored or disengaged.

Talented students possess distinct psychological traits that arise from the interplay between individual characteristics and their environment. Factors such as culture and opportunities also influence their development. These students may encounter challenges such as underachievement, low self-concept, and a lack of peer support. Consequently, the talent development framework places significant emphasis on deliberately fostering psychosocial skills that support high achievement, persistence, and creativity, all of which require motivation (Subotnik et al., 2011). To develop effective strategies that address this issue and promote positive outcomes, a better understanding of the experiences and needs of mathematically gifted students is compelled for adequate development of their creativity and talent (Barraza-García et al., 2020).

#### Wasted time and waiting

Waiting can be particularly tedious and uninteresting for gifted students, especially during their adolescent years. Gifted students experience three kinds of waiting in classrooms: school/classroom, instructional, and assignment (Coleman, 1997; Peine, 2003; Coleman and Cross, 2005; Peine and Coleman, 2010). *Classroom structure waiting* is a phenomenon that is connected to the organization of the classroom, as well as behavior and classroom shared characteristics such as rules or the location of student desks. *Instructional waiting* occurs when new material is presented, but gifted students are already familiar with it or learn it faster than others due to their advanced abilities. It involves the instructional model used by the teacher within the school system’s evaluation framework. *Assignment waiting* is the portion of the instructional period that is for classwork, workbooks, or homework. It occurs during a class that is scheduled for extended practice, or classwork, after the introduction of new concepts. Three conditions contribute to assignment waiting; gifted students finishing all assigned work at a faster rate than other students, students who complete classwork and homework assignments and forget to bring a book to read, and students who constantly anticipate the end of the day.

A qualitative study of 16 children in elementary and middle school revealed that sitting and waiting was a universal ingredient of being intellectually gifted; this grounded theory study revealed variations in context and actions, and implications for teaching, teacher evaluation, and classroom management. Statistically, among any group of students doing the same lesson, a few will learn faster and finish, several will not finish, and others take the amount of time the teacher intended. The range of achievement in a typical grade is more than 5 years, and gifted children arrive in class at the beginning of the school year knowing 40–60% of the content. Therefore, children who are gifted experience high levels of waiting that leads to boredom (Gagné, 2005; Peine and Coleman, 2010).

### Meaningful, enriched learning at an adequate level

The implementation of strategies based on the MAC model, alongside ability grouping, may create an enriched learning environment tailored to the abilities of gifted students. This would involve fostering experiences that align with students’ individual interests, needs, and pace of learning. This combination of strategies has the potential to protect gifted youth from experiencing boredom, as highlighted in a study by Feuchter and Preckel (2021). In addition, ability grouping is an easier and faster strategy to provide them with opportunities to go beyond the regular curriculum without hindering school administration. Most importantly, it improves academic performance, social skills, and creativity, all of which are important in preparing adolescent students for a healthy adult life (Valadez et al., 2020).

Prolonged boredom may reduce intrinsic motivation for learning in the long run if meaning is lost. However, by recognizing boredom as an alert sign, gifted students can leverage it to their advantage by assessing the engagement and meaning of the task at hand. This self-awareness enables them to make informed decisions about whether to invest their efforts and motivation in the task or seek more stimulating and meaningful alternatives.

To prevent boredom, teachers should design tasks that provide an individually optimal level of stimulation, which requires knowledge of students’ ability level and learning preferences (Acee et al., 2010; Preckel et al., 2010; Westgate and Wilson, 2018; Feuchter and Preckel, 2021). According to Preckel et al. (2010) and Feuchter and Preckel (2021), grouping gifted students by ability may have positive academic outcomes.

### Case of Paraguay: the present study

Schools play a crucial role in creating opportunities for gifted students at various stages of talent development, while community-based organizations, universities, and cultural institutions can offer valuable out-of-school programs (Olszewski-Kubilius and Clarenbach, 2012; Peters et al., 2014). In developing countries such as Paraguay, where no gifted and talented education programming exists and provisions for advanced learners are scarce, out-of-school programs take a more prominent role; as students might frequently encounter boredom in educational activities due to a mismatch of meaning and attentional components.

Using a sequential explanatory mixed method design, this study investigated boredom in exceptionally talented adolescents participating in an elite mathematical talent development program in Paraguay.

Research questions were as follows:

RQ1. Are exceptionally talented students in mathematics in Paraguay more prone to boredom than their age peers?

RQ2. Is boredom related to school attitudes and psychological well-being in this population?

RQ3. What factors impact their perception of boredom?

## Method

### Design

We used a sequential explanatory mixed method design (QUAN🡢QUAL) with a descriptive-exploratory quantitative phase and a phenomenological qualitative phase. Quantitative measures were taken using psychometric questionnaires; qualitative data was collected in focus groups to make meaning of the social experience of boredom and complement the quantitative data.

#### Phase 1: quantitative

##### Participants

In the quantitative phase, 54 adolescents between the ages of 12 and 17 participated in the context of a larger project on talent development in the year 2022 (*M* = 14.4 years, *SD* = 1.35). They were invited as part of an exclusive program for the development of exceptional mathematical talent, for which they were invited after reaching the finals in the National Math Olympics. They had been participating in the National Math Olympics for a mean of 5.17 years (SD = 2.30), ranging from the first year competing to the ninth year in the Olympics. As for participation in the mathematics talent development program, the mean was 2.15 years (SD = 1.57) with a minimum of 0 years (i.e., just beginning) to 6 years in it (i.e., about to graduate high school). Regarding gender distribution, 28 identified as girls (51.9%), 23 as boys (42.6%), one as non-binary (1.9%), and two preferred not to disclose gender (3.7%).

##### Instruments

Participants completed a demographic information form which asked for gender, age, city, schooling, years participating in the Math Olympics, and years participating in the enrichment program. They also completed three psychometric questionnaires:

###### Boredom Short Scale

In Spanish Escala de Aburrimiento (EsAb; Gonzalez Ramirez et al., 2021), it is a brief 7-item questionnaire in a 5-point Likert scale, developed in Mexico. It has two subscales: tendency toward boredom with four items, and lack of interest with three items. All items are positively worded.

###### Spanish adaptation of the School Attitude Assessment Survey-Revised

The SAAS-R is a 35-item questionnaire in a 7-point Likert scale (McCoach and Siegle, 2003). It has 5 subscales: attitudes toward school, attitudes toward teachers, academic self-perception, goal valuation and motivation/self-regulation; it was adapted with Spanish adolescents by Miñano et al. (2014). For this study, participants were asked to complete each item twice: once thinking about their school, and once thinking about their classes at the elite mathematical talent development enrichment program. In this way, we could test their perceptions and attitudes regarding formal schooling as well as the specific talent development program.

###### Psychological Well-Being Scale for Youth

In Spanish Escala de Bienestar Psicológico para Jóvenes (BIEPS-J; Casullo and Castro Solano, 2000), it is a brief 13-item questionnaire in a 3-point Likert scale, all positively worded. The BIEPS-J has four subscales: self-control with four items, interpersonal relationships with three items, personal projects with three items, and self-acceptance with three items. All items can be added for a general psychological well-being score, with a higher score representing higher well-being. Originally developed in Argentina, researchers in Paraguay have already used the BIEPS-J with strong reliability (Mc Donald’s *ω* > 0.80; Vuyk et al., 2023); similarly, Mexican researchers also found satisfactory psychometric properties (Cortez Vidal, 2016).

##### Quantitative data analysis plan

To analyze quantitative data, we first conducted descriptive analyses. In addition, we conducted inferential analyses to compare the study sample to the general population, using reference data from Mexican adolescents in the validation study by Gonzalez Ramirez et al. (2021). For these comparisons, we performed independent mean difference estimation analyses (Calin-Jageman and Cumming, 2019). This analysis incorporates the calculation of *t*-tests for independent samples with an estimation of differences, calculating effect sizes and 95% confidence intervals, to assess whether differences are statistically and practically significant. Thus, the hypothesis test does not simply put a cut-off point to mark differences as in *t-*tests, but estimates the size of the difference and the uncertainty around it.

#### Phase 2: qualitative

##### Participants

For Phase 2, we used purposive sampling to reach mathematically talented students as they represented the population of interest. All students in the mathematics enrichment program (*N* > 100) were invited to participate in focus groups as part of a larger project on talent development in the year 2023, and 50 participated. Their ages ranged between 15 and 18 (*M* = 15.8 years, *SD* = 1.05). Seven focus groups of six to 11 students with exceptional mathematical talent were conducted by project researchers, previously trained in the methodology. Students were randomly assigned to groups, and group sessions lasted between 40 to 60 min.

##### Instruments

A semi-structured questionnaire served as a guide to gather information about the experiences of boredom among students in focus groups. This questionnaire was developed by the research team, based on the MAC model of boredom and relevant literature on boredom in gifted and talented students in general. The questions included whether they considered school is boring, what bores them about school, what they think about when they are bored, their levels of boredom during school, what usually entertains them, whether their parents try to keep them busy at home, whether it is hard for them to get out of boredom, and what they do to overcome boredom. This questionnaire provided a structured approach to understanding the experiences of boredom among students, allowing for the identification of common themes and patterns.

##### Qualitative data analysis plan

Qualitative data was analyzed using a thematic analysis approach (Braun and Clarke, 2006). First, researchers transcribed and read through interview data to become familiar with the content. Next, the first author generated initial codes; these were developed inductively from the data and aimed to capture the meaning of the text. Initial codes were then grouped together to form potential themes that captured the key ideas and concepts within the data. The second author reviewed the preliminary list of themes and categories, providing an additional layer of verification for the thematic analysis. To ensure the rigor and validity of the thematic analysis, constant comparison was used to develop themes that would reflect participants’ insights. This process involved comparing and contrasting categories to identify similarities and differences, ultimately leading to the identification of overarching themes. Finally, all three authors reviewed the themes and categories, along with the main findings of the study, to further ensure the validity of the results.

##### Researcher description: reflexivity statements and experience

We are female researchers in a developing country, with lived experience of boredom in adolescence leading to a waste of time and motivation as talented students. One of the researchers participated in mathematical talent programs, while the others did not. We acknowledge that our prior understanding of the phenomenon of boredom may be influenced by our cultural background and personal experiences. We recognize the potential for our own biases to shape our interpretation of the data and analysis. We strived to maintain reflexivity throughout the research process by constantly reflecting on our assumptions, beliefs, and perspectives, and by seeking feedback from participants and other researchers in the field. Through this process, we hope to ensure that our findings accurately reflect the experiences and perspectives of the gifted students in this study and contribute to a more nuanced understanding of the phenomenon and potential consequences of boredom in this population.

###### Triangulation of results: the main strength of mixed methods

By combining both qualitative and quantitative data, we gained a more comprehensive understanding of the phenomenon under investigation. In our study, we used triangulation to complement and contextualize our findings, utilizing the breadth of quantitative data and the depth of qualitative data. This allowed us to explore the phenomenon from multiple angles, using both numerical data to identify trends and patterns, and qualitative data to provide insights into the experiences and perspectives of participants. In the triangulation of our results, we were able to develop a more detailed and comprehensive understanding of the phenomenon of boredom.

## Results

### Quantitative analyses

This section presents statistics for boredom in Paraguayan adolescents with exceptional mathematical talent, along with additional relevant data to aid in interpretation of results.

#### Boredom patterns in mathematically talented adolescents

To answer RQ1, “Are exceptionally talented students in mathematics in Paraguay more prone to boredom than their age peers?” this section presents descriptive statistics for boredom in this sample, along with comparisons to the reference group in the original instrument validation study.

Table 1 exhibits the means, standard deviations, estimated independent mean differences, and effect sizes of subscales and items of the Boredom Short Scale for the quantitative study sample of 54 participants as well as the original validation sample of 600 Mexican adolescents (Gonzalez Ramirez et al., 2021).

**Table 1:** Means, standard deviations, estimate independent mean differences and effect sizes of the Boredom Short Scale in the study sample (*N* = 54) and the original validation sample (*N* = 600).

		Study sample		Validation sample		Estimate Independent Mean Differences			
		(*N* = 54)		(*N* = 600)		(*df* = 652)			
		**Mean**	**SD**	**Mean**	**SD**	***t***	***p***	***d***	**95% CI**
Subscale 1: Tendency toward boredom		1.87	1.00	1.64	0.99	−1.67	0.096	−0.24	[−0.52, 0.04]
1.I stop doing activities because I get bored [Dejo de hacer actividades porque me aburro]		2.04	1.24	1.70	0.88	−2.62	0.009	−0.37	[−0.65, −0.09]
2.I get bored easily [Me aburro con facilidad]		2.15	1.29	1.85	1.04	−1.99	0.047	−0.28	[−0.55, −0.00]
3.Everything seems repetitive and routine to me [Todo me parece repetitivo y rutinario]		2.13	1.27	1.80	1.11	−2.07	0.039	−0.29	[−0.57, −0.01]
6.Everything seems boring to me [Todo me parece aburrido]		1.17	1.24	1.19	0.94	0.15	0.884	0.02	[−0.26, 0.30]
Subscale 2: Lack of interest		1.23	1.05	1.49	1.04	1.75	0.080	0.25	[−0.03, 0.53]
4.Few things catch my attention [Pocas cosas me llaman la atención]		1.39	1.20	1.54	1.09	0.96	0.336	0.14	[−0.14, 0.42]
5.It’s hard for me to get excited about something [Es difícil que algo me entusiasme]		1.19	1.20	1.49	1.02	2.03	0.043	0.29	[0.01, 0.57]
7.It’s hard for me to get interested in something [Es difícil que algo me interese]		1.13	1.15	1.44	1.02	2.12	0.034	0.30	[0.02, 0.58]

The validation sample comes from the original article by González Ramírez et al. (2021).

Reliability estimates in the study sample are good for the total Boredom Short Scale (McDonald’s *ω* = 0.884) and the two subscales, Tendency Toward Boredom (McDonald’s *ω* = 0.808) and Lack of Interest (McDonald’s *ω* = 0.869). These reliability coefficients are higher than subscale reliability estimates in the validation study of Cronbach’s *α* of 0.695 and 0.725, respectively (Gonzalez Ramirez et al., 2021). Subscales present a strong direct correlation of *r* = 0.69 (*p* < 0.001).

Table 1 shows the mean and standard deviation for each item on the subscales, as well as the estimated independent mean differences and effect sizes between the study sample and the validation sample in Gonzalez Ramirez et al. (2021). The table also provides the *t*-value and *p*-value for each mean difference, along with the 95% confidence interval for the effect size.

Boredom shows a different pattern in mathematically talented adolescents compared to the validation sample. On one hand, subscales with composite scores do not show significant differences. However, item distributions within those subscales differ significantly.

In the subscale of Tendency toward Boredom, the composite score does not differ, but in three of the four items the mathematically talented students score higher than the population average; those items are “*I stop doing activities because I get bored*,” “*I get bored easily*,” and “*Everything seems repetitive and routine to me*.” The item “*Everything seems boring to me*” did not differ among the mathematically talented adolescents and the general adolescent population.

On the composite score for the Lack of Interest subscale, mathematically talented students scored like the general adolescent population; however, on two of the three items they scored lower than the general population. This is a reversed pattern compared to the items in tendency toward boredom. The items in which mathematically talented adolescents score lower are “*It’s hard for me to get excited about something*” and “*It’s hard for me to get interested in something*.” No differences existed in the item “*Few things catch my attention*.”

#### Factors related to boredom: correlations with school attitudes and psychological well-being

To answer RQ2, “Is boredom related to school attitudes and psychological well-being in this population?” we conducted Pearson correlations among subscales of boredom, attitudes both at their school and the mathematical enrichment program, and psychological well-being. Table 2 shows intercorrelations among variables, and we will highlight the most relevant.

**Table 2:** Intercorrelations among boredom, school attitudes and psychological well-being (*N* = 54).

		**1**	**2**	**3**	**4**	**5**	**6**	**7**	**8**	**9**	**10**	**11**	**12**	**13**	**14**	**15**	**16**	**17**	**18**	**19**	**20**	**21**
1	EsAb: total	—																				
2	EsAb: tendency toward boredom	0.938***	—																			
3	EsAb: lack of interest	0.896***	0.687***	—																		
4	SAAS-R: attitudes toward teachers at school	–0.323*	–0.215	–.402**	—																	
5	SAAS-R: attitudes toward teachers at the program	–0.365**	–0.325*	–0.349**	0.256	—																
6	SAAS-R: attitudes toward school	–0.398**	–0.308*	–0.441***	0.797***	0.283*	—															
7	SAAS-R: attitudes toward the program	–0.354**	–0.315*	–0.340*	0.187	0.758***	0.382**	—														
8	SAAS-R: goal valuation at school	–0.183	–0.191	–0.140	0.612***	0.370**	0.509***	0.196	—													
9	SAAS-R: goal valuation at the program	–0.260	–0.327*	–0.126	0.360**	0.528***	0.300*	0.391**	0.743***	—												
10	SAAS-R: motivation and self-regulation at school	–0.374**	–0.378**	–0.301*	0.621***	0.292*	0.609***	0.192	0.744***	0.498***	—											
11	SAAS-R: motivation and self-regulation at the program	–0.497***	–0.462***	–0.450***	0.304*	0.524***	0.341*	0.396**	0.500***	0.553***	0.666***	—										
12	SAAS-R: academic self-perception at school	–0.006	0.014	–0.030	0.323*	0.220	0.112	0.067	0.341*	0.153	0.437***	0.269*	—									
13	SAAS-R: academic self-perception at the program	–0.267	–0.283*	–0.199	0.077	0.390**	0.105	0.285*	0.110	0.302*	0.330*	0.698***	0.350**	—								
14	BIEPS-J: self-control	–0.370**	–0.354**	–0.321*	0.018	0.179	0.114	–0.175	0.145	0.191	0.240	0.494***	0.130	0.481***	—							
15	BIEPS-J: interpersonal relationships	–0.129	–0.012	–0.255	0.177	0.094	0.294*	0.196	–0.080	–0.104	0.018	0.091	–0.055	0.180	0.197	—						
16	BIEPS-J: personal projects	–0.358**	–0.330*	–0.329*	0.198	0.076	0.119	0.095	0.251	0.291*	0.415**	0.498***	0.329*	0.375**	0.402**	0.119	—					
17	BIEPS-J: self-acceptance	–0.124	–0.160	–0.056	–0.089	0.032	0.090	0.097	–0.152	–0.032	0.006	0.228	–0.131	0.550***	0.535***	0.349**	0.036	—				
18	BIEPS-J: general psychological well-being	–0.377**	–0.338*	–0.358**	0.101	0.146	0.213	0.204	0.079	0.149	0.265	0.508***	0.115	0.598***	0.836***	0.550***	0.593***	0.707***	—			
19	Age	0.032	–0.053	0.134	–0.518***	–0.069	–0.366**	0.075	–0.278*	–0.167	–0.344*	–0.206	–0.141	–0.071	0.028	0.021	–0.108	–0.020	–0.028	—		
20	Years in math Olympics	–0.008	–0.038	0.032	0.021	–0.118	–0.024	–0.248	0.197	–0.030	0.147	–0.071	0.060	–0.190	0.065	–0.001	0.114	–0.142	0.020	0.261	—	
21	Years in the program	0.085	0.000	0.177	–0.436***	0.038	–0.314*	0.059	–0.126	–0.088	–0.259	–0.153	–0.232	–0.130	0.060	0.012	–0.157	0.056	–0.008	0.577***	0.495***	—

*p < 0.05, **p < 0.01, ***p < 0.001.

##### Boredom and school attitudes

Correlations between boredom variables and school attitudes in mathematically talented students depend on the context; some are similar for the enrichment program and their school, yet others differ. Years of participation in the enrichment program and the Math Olympics, as well as age, are unrelated to boredom.

Attitude toward teachers at school is inversely related to lack of interest (*r* = −0.402, *p* < 0.001) but not tendency toward boredom. This indicates that the mathematically talented students that find it more difficult to get excited about things have a worse attitude toward their school teachers, yet do not tend to get stuck in routines. At the mathematical enrichment program, attitude toward teachers has a small inverse correlation with lack of interest (*r* = −0.349, *p* < 0.01) and tendency toward boredom (*r* = −0.325, *p* < 0.05). Thus, students with a more negative attitude toward their program teachers find it difficult to spark their interest and are quicker to stop doing things due to boredom. Attitudes toward school and toward the enrichment program are moderately related to both tendency toward boredom and lack of interest, all with inverse correlations ranging between *r* = −0.308 and *r* = −0.441.

Goal valuation is not related to tendency toward boredom or lack of interest at school; mathematically talented students likely value goals regardless of becoming easily bored or interested. However, at the enrichment program, where all activities are more goal-focused, there is a small inverse correlation between goal valuation and tendency toward boredom (*r* = −0.327, *p* < 0.05) but not lack of interest. They get interested independent of whether they value their goals at the program or not; students with lower goals at the program tend to get bored and quit easier, and vice versa. In fact, at the enrichment program, motivation and self-regulation have a stronger inverse relationship with tendency toward boredom (*r* = −0.462, *p* < 0.001) and lack of interest (*r* = −0.450, *p* < 0.001) than at school (*r* = −0.378, *p* < 0.01 and *r* = −0.301, *p* < 0.05, respectively).

Academic self-perception is not associated with boredom at school; mathematically talented students see themselves as academically capable whether they get easily bored or find additional interests. Nevertheless, academic self-perception shows a small inverse correlation with tendency toward boredom at the mathematics enrichment program (*r* = −0.283, *p* < 0.05). Therefore, mathematically talented students who stop activities due to getting easily bored tend to see themselves as less capable in the program and vice versa.

##### Boredom and psychological well-being

Regarding general well-being in mathematically talented students, there is an inverse relationship with tendency toward boredom (*r* = −0.338, *p* < 0.05) and lack of interest (*r* = −0.358, *p* < 0.01). Self-control is inversely related to both tendency toward boredom (*r* = −0.354, *p* < 0.01) and lack of interest (*r* = −0.321, *p* < 0.05). On the other hand, personal projects are also inversely related to both tendency toward boredom (*r* = −0.330, *p* < 0.05) and lack of interest (*r* = −0.329, *p* < 0.05). However, interpersonal relationships and self-acceptance do not show any correlations with boredom.

### Qualitative analyses

**Figure 1:**
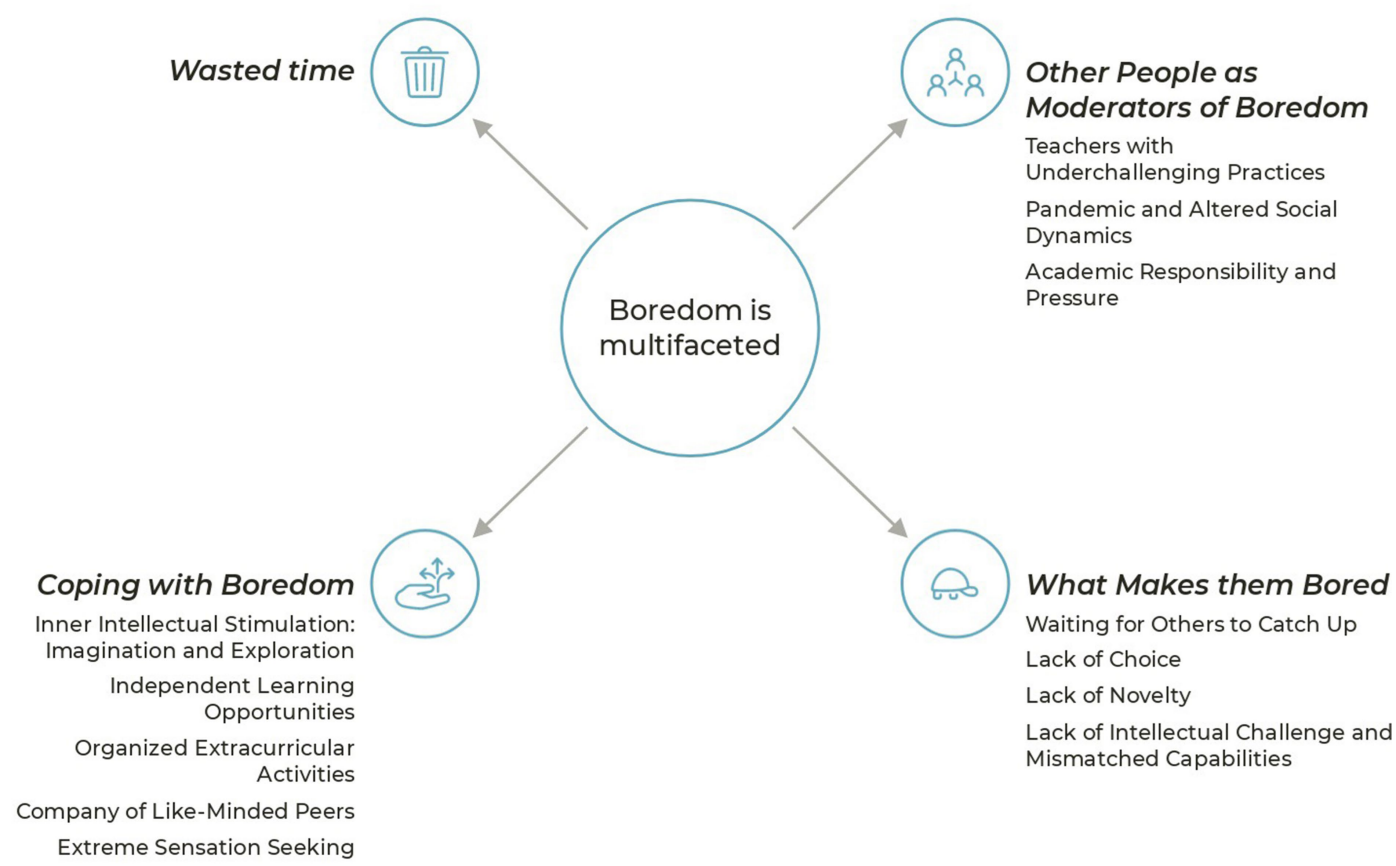
Thematic map of boredom and perception of related factors in mathematically talented adolescents.

To answer RQ3, “What factors impact their perception of boredom?,” we present themes and categories developed in the thematic analysis based on data gathered in focus groups (see Figure 1), providing illustrative quotes from participants to support the analysis. We present a framework for examining mathematically talented students’ perception of factors that influence their experience of boredom.

The central theme developed refers to the multifaceted nature of boredom experienced by mathematically talented students; we explored dimensions of boredom, including a lack of deep meaning, lack of superficial entertainment, wasted time, and the factors that contribute to boredom such as waiting for others, lack of choice, lack of novelty, lack of intellectual challenge, and the role of teachers and social dynamics. Additionally, the text discusses how mathematically talented students cope with boredom through strategies such as inner intellectual stimulation, entertainment and escape, independent learning opportunities, organized extracurricular activities, company of like-minded peers, and even extreme sensation seeking.

#### Multifaceted (confusing?) concepts of boredom

The use of the term “boredom” by students reveals varying meanings and nuances in their emotional experiences. This section delves into the different dimensions of boredom expressed by mathematically talented students, ranging from a deeper lack of meaning to a superficial absence of entertainment (e.g., *“not all boredoms are the same, and not all of them are easy to kill”*). Mathematically talented students used the word boredom in at least two meanings: one, boredom as a lack of meaning in a deeper sense (e.g., *“It’s like you cannot get over your boredom, because you are still bored watching TikTok”; “there are times when you think of something to do but you feel helpless that you cannot do it and you do not do it in the end because of your helplessness, because of your fear of failure”*) and the other, as a superficial lack of entertainment (e.g., *“When I’m at home I do not have much to do so I watch series and often those emotions rub off on me”*).

The interchangeability of these connotations suggests potential confusion and the coexistence of multiple interpretations of boredom among mathematically talented students; “*“If it’s a short boredom, I use my cell phone, but if it’s more of a deeper boredom, I do think I need to do something more extreme.”*

In addition, inconsistencies in emotional descriptions and incorrect labeling of situations hint at a lack of clear understanding regarding the accurate categorization of emotions. *“I do not know if that’s boredom, I would say lack of motivation. Sometimes not knowing what to do leads me to a lack of motivation. I do not know if they are partly related.”*

#### Wasted time

Mathematically talented students expressed concerns about feeling unproductive when they had nothing to do, highlighting the importance they place on utilizing their time effectively. *“I’m a person who spends a lot of time doing activities with a lot of responsibilities, and I do not know, I feel like I’m wasting my time and I can do much more productive things at that time. I think about, when is this going to end? I have a lot of things to do that I feel are much more important and I’m wasting my time, basically.”*

They expressed a strong aversion to idleness, stating that they become stressed when they perceive their time as being wasted. *“Normally, I get stressed out when I have nothing to do, I feel like it’s time wasted. If I get bored I look on YouTube for something intellectually stimulating.”* Mathematically talented students want to be constantly engaged in activities that they deem valuable. *“I do research by myself and all of that, and it seems like a waste of time what I’m doing in school.”*

This mindset reflects their high motivation and drive to make the most of their time, showing their goal-oriented approach to learning and personal growth. *“I’m focusing a lot on what my bachelor’s degree would be, but they [at school] wander a lot, they waste a lot of time. They focus a lot more on other things than on what should be, instead of taking advantage of the time and learning more...”*

#### What makes them bored

##### Waiting for others to catch up

Waiting for others to catch up was a common source of boredom for mathematically talented students, since it frequently indicated that they were not being challenged at an appropriate level for their ability. *“On Wednesday to be exact I had an accounting assignment due in 3 weeks, and I finished it to be exact on Thursday, it took me 8hs and 19 sheets of my notebook. To this day I am still bored because I have no homework to entertain me. The same in mathematics, reviewing...the same with the other classes, the same, nothing new, nothing that interests me right now.”*

They reported that they often acquired and assimilated material faster than their peers, and therefore became bored and disinterested when obligated to wait for others to catch up to their level of understanding (e.g., “*I hate it when you have to wait too long for something so easy*”). This seemed to be especially irritating in the general education classroom in their schools, rather than in the mathematics enrichment program.

In school, teachers expected the mathematically talented students to work at the same rate as their classmates, even if they could finish the job faster*; “there are times when you cannot do anything, for example at school, you have done everything, you are bored and you want to use your cell phone but they will not let you.”* Boredom, disengagement, and even dissatisfaction or resentment seemed to result from this.

##### Lack of choice

Not being able to choose what to pursue was mentioned as problematic; mathematically talented students wanted the opportunity to explore new and challenging topics or pursue their own interests. In traditional settings they could not do so, as memorization was prioritized and classes were rarely stimulating (e.g., “*when the environment is not nice, when you get bored easily, when the teacher does not know how to teach or when to teach, it is way too boring*”), without sufficient opportunities for challenge and growth.

Mathematically talented students mentioned frequently that when they could choose activities, the level of boredom decreased (e.g., *“It’s nice to do things by yourself, being told what to do makes the task boring*”). The majority discussed the importance of choosing useful or relevant activities at certain times, such as when studying for international Math Olympics or scholarships.

Having to do the same activities as the other students, regardless of level of ability or achievement, was reported to be frustrating; this was the case especially when mathematically talented students perceived that they did not have any control over assigned tasks. *“At school is where I get bored the most because I have no options, I cannot do anything, I cannot get up, I cannot take out my computer or do other homework, so you have to defend yourself with what you have at hand, try to write something, I do not know, I do not know, I do not know well. It’s kind of complicated and annoying. In other activities or places you have more options of what things you can do to have fun and be busy, anything, but not at school.”*

##### Lack of novelty

Repetition and routine were perceived as potential causes of boredom, particularly when mathematically talented students were being forced to spend a lot of time on material that was too easy or repetitive without any new perspectives; “*in mathematics, our teacher usually uses a whole month to explain, or in English too, 50 times the same fact, the same causes and consequences, and that is very boring, when the same thing is always repeated.”*

In their quest for entertainment and engagement, mathematically talented students highlight the importance of breaking the monotony of repetitive activities. They recognize that even enjoyable tasks can become boring if they lack variety and new experiences. Mathematically talented students seek opportunities to diversify their activities, introducing novelty and fresh perspectives to sustain their interest and motivation. *“Novelty is what entertains me. I generally enjoy trying new things, looking at the limit of things and challenging myself to see my ability or to improve, to try what I like, to have new activities where I can develop myself more, where I can acquire new skills.”*

##### Lack of intellectual challenge and mismatched capabilities

Insufficient intellectual challenge was pervasive in their school settings. Mathematically talented students indicated not feeling like they were learning anything new or challenging their abilities in school; they reported schoolwork to be “*simple and irrelevant,*” with tasks that “*do not require logic*.” They reported being bored because the tasks or subjects they encountered were not challenging or stimulating enough for their capabilities and interests; *“It does not make me work hard, so it bores me;” “What bores me is that it is not a challenge for the capacity that I have, in most subjects....the subjects that challenge me, both psychologically and physically, are subjects where we have to do research, those stimulate me and that is what I like.”*

Challenge was present in the math enrichment program, yet perceptions of challenge levels varied in mathematically talented students from rural vs. urban areas. In the math enrichment program, talented students from the countryside found classes too fast-paced and stated it was challenging to keep up with the workload (e.g., “*They give you the problem sheet, but you cannot solve it right away like the others*”). Mathematically talented students from urban areas, especially from the major metropolitan area in Paraguay, considered challenge levels adequate and reported little or no boredom. They attributed this to the variety of activities offered in the program and the motivation they received from peers to engage with activities.

#### Other people as moderators of boredom

##### Teachers with underchallenging practices

Teachers’ impact on mathematically talented students’ perceptions of school and boredom seems to be a key factor for boredom. These mathematically talented students reported needing dynamic and exciting teaching to boost their interest. Healthier relationships with teachers helped them engage in school more effectively. They described their school teachers as being too old-fashioned and/or not focusing on what students wanted to learn.

While mathematically talented students generally found subjects interesting, when teachers were not engaging they reported getting bored (e.g., “*for example, my history teacher, I like that subject, but he never gave a good class, he would say copy this, do this, and never explained anything, so I did not enjoy the class, even though I was interested in it”*). Similarly, when subjects were monotonously and halfhearted taught, mathematically talented students lost all interest in them. Many negative experiences with teachers were brought to attention. This included teachers who did explain mathematical problems well because they did not provide enough information. Other teachers did not seem committed to the subject matter. Teachers who were traditional in the way they teach, not approaching subjects in depth and only superficially, were also mentioned as sparking boredom. More severe experiences included teachers who ridiculed or disregarded students’ viewpoints (e.g., *“In math classes I usually ask a lot of questions like where equations come from and teachers tell me to just shut up, and that sucks”*).

##### Pandemic and altered social dynamics

Social interaction played an important role in the experience of school and boredom. Mathematically talented students mentioned that the pandemic had affected their experience at school and had left some students feeling lonely due to the loss of friends (e.g., *“When the pandemic came, most of my friends left the program and did not come back. So, it happened to me many times that I was very lonely”*).

In addition, disruptions to normal routines and changes in the learning environment seemed to have led to a sense of disorientation and lack of motivation for some of the mathematically talented students. Others complained that their classmates were too chaotic and rebellious, which they found annoying and disruptive; it became a hindrance to their own learning and academic progress. Finally, some of the mathematically talented students student’s felt frustrated when their classmates did not share their interest in learning or did not take their academic responsibilities seriously, which seemed to create a sense of dissonance and disconnection from their peers. “*When you stand out in your group they tend to throw all the responsibility at you*.”

##### Academic responsibility and pressure

Some mathematically talented students highlighted the burden of academic responsibility they experienced when standing out within their group. They expressed frustration when classmates did not share the same level of dedication to learning or failed to take their academic responsibilities seriously. This imbalance in the distribution of responsibilities created a sense of unfairness and added pressure on these students. Nevertheless, they had different opinions regarding leadership and the social pressure that may surround it as well. Many reported they felt the same school pressure to perform well and live up to high expectations in their extracurricular activities. Additionally, on occasions they felt specific social pressure from adults like parents, mentors, and teachers, which contributed to a sense of pressure or stress, as one student states: *“People place a burden on me with a lot of things: being a class representative, organizing and stuff like that. Also, I work in the family business, so I get home, shower, change and go to the business to work and that’s part of my routine.”* Others do not feel comfortable with leading roles itself: “*I do not like leading so much, I think it’s something admirable and that if the person likes it they can improve a lot of things. But I do not like to lead, I just want to be part of the group, do my part, contribute and that’s it*.”

#### Coping with boredom

Mathematically talented students reported they went out of their way to escape boredom and find entertainment. However, not all strategies yielded the desired effectiveness; *“If I’m just bored at that moment, but emotionally stable, I can do anything [to get out of boredom], but if I’m tired or something has me down, it’s harder for me to find something.”* While some strategies predominantly offered relief from boredom, others required additional regulation to serve their intended purpose in a healthy manner, and some strategies even carried the potential for risk behavior patterns. The following strategies were most common:

##### Inner intellectual stimulation: imagination and exploration

Mathematically talented students often used inner intellectual stimulation as a coping mechanism against boredom. Some mathematically talented students only needed their imagination to wander in creative scenarios, thoughts, and ideas; *“I am very distracted, anything, a line on the table already makes me think of a million things, that’s when it starts. I start thinking about what it can be or what I can do with those lines. And then I think about games and how to save coins in the game, anything.”*

##### Entertainment and escape

Both inside and outside of school, mathematically talented students mentioned they liked to listen to music or read books. *“To combat boredom I bought 7 books but if there is some part that simply bores me I wander into another thought, I read the book but at the same time I am thinking about something else. The same thing happens to me in class, like the teacher is giving his class I think of something else or in social situations for example I usually think of many physical mechanisms of things or also deduce the behavior of people.”* Specific forms of entertainment outside of school were playing sports, reading comics, drawing, and socializing with friends. Specific strategies used in school were using electronic devices, drawing, reading, and daydreaming.

##### Independent learning opportunities

In the classroom, mathematically talented students tended to advance in homework or activities by turning to independent study to stimulate their intellectual curiosity and maintain their engagement with learning (e.g., “*If I have something to do in the afternoon, I try to get ahead with schoolwork, I try to think about organizing myself further*”). This included reading, listening to podcasts, watching YouTube channels or other videos about their topics of interest. Learning by themselves allowed them to choose their own topics, set their own goals, explore in depth and at their own pace, and work at a level appropriate for their abilities.

Oftentimes, mathematically talented students used these independent learning opportunities to keep themselves intellectually engaged, especially when they felt unchallenged in their regular coursework. *“I try to make some time to do what I like, such as looking at articles about physics, neuroscience, things like that, watching videos. I try to organize some time between homework and these activities that I have to do, for example I was learning the methods to put together the [Rubik] cubes, like the bigger ones, and programming.”* By doing so, they maintained intellectual curiosity and passion, even amid an educational environment that might not cater to their individual interests and abilities.

##### Organized extracurricular activities

Mathematically talented students participated in varied extracurricular activities such as skate, golf, basketball, football, gymnastics, violin, language studies, lettering, and programming. These extracurricular activities allowed them to explore and pursue their intense interests and passions in structured and supportive environments outside of school (e.g., *“I do not get bored with basketball, I like it because it’s very varied and we usually change the things we do,”* “*I have more fun in extracurricular classes than in school, because I do what I enjoy*”). Although some felt pressured to balance their academic workload with their extracurricular activities, they generally valued these activities because it offered them opportunities for leadership, creativity, and personal growth.

##### Company of like-minded peers

Mathematically talented students at the enrichment program found in peers who shared their interests and/or abilities, a sense of community and motivation to pursue their interests. According to their comments, peer relationships took many forms; including participating in extracurricular leisure activities together (e.g., skating, golfing, video gaming, etc.), attending specialized programs or events, and forming school groups; e.g., “*Being with my friends, the boredom is gone*.”

Some of the mathematically talented students mentioned they sought company and felt good around kind people even if they did not share the same interests. Others mentioned that laying in the grass, spending time with a partner, or meeting new people during break at school provided them with a sense of belonging and social connection. It also helped them to relieve stress and engage in activities that were not necessarily related to academic achievement but were beneficial to their overall well-being and helped them cope with boredom.

##### Extreme sensation seeking

When some mathematically talented students felt bored at school, they tended to engage in what they called *“exciting*” activities, probably drawn by extreme sensation seeking; the desire to engage in thrilling, high-risk activities to experience an adrenaline rush or a sense of excitement. An example is *“To get out of boredom I need strong experiences like the time when there was 25-meter deep water well and I jumped in and I was never going to touch the bottom, that was fun and exciting.”*

When mathematically talented students were bored at school, on occasions they turned to disruptive behavior to alleviate their boredom. While these activities may have provided a temporary sense of excitement, students recognized they could also be harmful and have long-term negative consequences (e.g.; *“several times we went too far,” “it is no longer fun when they throw things in my face”*).

### Triangulation of results

Mathematically talented students in this study reported to have a relatively easy time discovering their interests, but they tend to disengage earlier than their counterparts in the general adolescent population when they become bored. These mathematically talented students experienced boredom more easily than their peers due to the ease and repetitiveness of tasks. However, they were adept at identifying and implementing strategies to alleviate their boredom; *“I end up thinking by myself and I’m already unbored.”* We sought to understand their subjective experience of being bored as well as their perception of factors impacting boredom.

When mathematically talented students lost interest, their opinion of teachers at school tended to decline. This could be due to factors that contribute to boredom and disinterest, regardless of how they perceive their teachers. Mathematically talented students commonly express a sense of not being challenged enough and lacking personal interest in school subjects, which can lead to disinterest and a negative view of teachers, as they may blame them for not engaging them effectively in the classroom. This may also be related to the idea of boredom as a lack of meaning, which emerged during group discussions.

However, when talented students lost interest in the mathematical enrichment program, not only did their attitude deteriorate, but their levels of boredom also increased. This is likely because their engagement in the program becomes more crucial when school, in general, is perceived as uninteresting. “*At school everything it’s very methodical. The program is more playful, there’s a bigger vision of the problems, they make you look at them in different ways, they make you think more*.” Additionally, since they are accustomed to being challenged and stimulated in the program, they do not anticipate experiencing disinterest. “*Even though the program has a structure, which seems routine, no two Saturdays are ever the same, never ever, because of the theory, because of the teachers, it’s very cool*.”

Goal valuation and the tendency toward boredom at the enrichment program are inversely related. Mathematically talented students who demonstrate greater perseverance are more likely to place higher value on the program itself. This is probably associated with students’ sense of worth since the enrichment program is an elite structure in which they willingly participate and in which they try to stand out at a national level. *“What I enjoy are the conversations that arise from the people I’m with in the program, they are very fun, interesting and really exciting,” “There are times in the morning when I solve a problem and I am so excited that I want tell my friends at the program, I want to explain it to them.”*

In fact, at the enrichment program, motivation and self-regulation have a stronger inverse relationship with tendency toward boredom and lack of interest than motivation and self-regulation at school for mathematically talented students; e.g., “*It is very difficult to get the motivation to do work alone at home, but being at the program you exchange views and engage with your peers,” “In the program you see what other people are doing and then it motivates you to do it too,” “At home I get bored solving math problems but when I come here it’s great to do it with everyone.”* Moreover, while academic self-perception is not linked to boredom at school, it exhibits an inverse correlation with boredom in the program, which aligns with the accounts provided by students.

Psychological well-being is inversely related to both aspects of boredom, just like self-control is inversely related to both aspects of boredom in mathematically talented students. As they are more likely to have the motivation and discipline to seek out additional learning opportunities beyond what is offered in their regular school setting, students may be able to engage in learning activities that are more challenging or complex without feeling bored or frustrated, and thus they may not need to exert as much self-control (e.g., *“I love to do the accounting tasks, they are very extensive, there is a lot of number crunching and calculus which I love,” “I love physics and hard neuroscience stuff*”).

Personal projects are also inversely related to lack of interest and tendency toward boredom. Mathematically talented students with higher degree of personal projects tend to be less prone to boredom and tend to find many things exciting. Interpersonal relationships were positively highlighted in focus groups as a coping mechanism against boredom; however, this may not be reflected in test results considering the focus on cognitive measures of boredom. Self-acceptance was not related to boredom variables in mathematically talented students.

Demographic information such as age, years of participation in the program, and in the Math Olympics, were not related to any boredom variables in mathematically talented students. Comparisons between mathematically talented students and the average population revealed that mathematically talented students tend to score higher than the average in tendency toward boredom (see Table 3). However, they also tend to score lower than the population average in measures of difficulty getting excited or interested in things, suggesting that mathematically talented students may have a greater range of interests and be more easily engaged in stimulating activities.

**Table 3:** Integrated results matrix of quantitative and qualitative data on boredom in mathematically talented adolescents.

Quantitative results	Qualitative results	Exemplar quote
Talented students scored **higher** than the population average in “*Everything seems repetitive and routine to me*.”	Wasted time, lack of novelty	*“I started to get bored with dance because... all the things were the same around there... before we used to go to competitions that were like wow, you got to know people, you danced and it was fun, but now they are like, very repetitive classes”*
		*“I do not get bored with basketball, I like it because it’s very* var*ied and we usually change the things we do”*
Talented students scored **higher** than the population average in “*I stop doing activities because I get bored*”	Inner intellectual stimulation, extreme sensation seeking	*“What I try to do is to put aside what bores me, even if I have to stop paying attention to the teacher, I stop looking at him, I stop listening to him, I grab a notebook, I do what I have to do, what interests me”*
Talented students scored **higher** than the population average in “*I get bored easily*”	Wasted time, lack of novelty, lack of challenge, extreme sensation seeking	*“I have a lot of tasks where I have to make a lot of drawings and do a lot of pressure calculations, things like that which I find very basic and boring”*
		*“When you are bored, making a mess is the most entertaining thing to do”*
		*“If it’s a short boredom, I use my phone, but if it’s a deeper boredom, I need to do something more extreme.”*
		*“To get out of boredom I need strong experiences like the time when there was 25-meter deep water well and I jumped in and I was never going to touch the bottom, that was fun and exciting”*
Talented students scored **lower** than the population average in “*It’s hard for me to get excited about something*”	Inner intellectual stimulation, independent learning experiences, organized extracurricular activities	*“When I have to calculate, to think, to get to the result, it is more exciting than memorizing a lot”*
		*“I have fun when we have math class”*
Talented students scored **lower** than the population average in “*It’s hard for me to get interested in something*”	Inner intellectual stimulation, independent learning experiences, organized extracurricular activities	*“I am overcome by the desire to want to do more things. At the end of the day, it’s all worth it because I gained experience.*
		*“My interests are more intellectual and mathematical”*
Attitude toward school and teachers correlated with lower tendency toward boredom and lower lack of interest	Lack of challenge, impact of teachers	*“Having more time with technical teachers makes it more enjoyable to be at school because I have a full day with my common curriculum teachers like science and chemistry”*
Higher motivation and self-regulation correlated with lower tendency toward boredom and lower lack of interest, especially in the math talent development program	Independent learning experiences	*“I am no longer at that time when I was 12 or 15 years old when I could do what I liked, now I do only what is necessary”*
Higher self-control and personal projects correlated with lower tendency toward boredom and lower lack of interest	Inner intellectual stimulation, independent learning experiences	*“When there is something that does not call my attention, I do it and I look for the way to make it fit in my life and enjoy it.”*

## Discussion

In the context of the MAC model of boredom, we present triangulated results for our research questions. RQ1, “Are exceptionally talented students in mathematics in Paraguay more prone to boredom than their age peers?” was supported with significant differences among subscale items.

Mathematically talented students often experience a higher propensity for boredom, which can be attributed to low arousal levels and a lack of challenge or novelty in their academic environment. According to the MAC model, boredom arises when individuals perceive their current situation as lacking in meaningful engagement, stimulation, and arousal (Westgate, 2020). Mathematically talented students, as well as gifted and talented students in general, with their advanced abilities and greater intellectual capacity, may find regular classroom activities and assignments to be less stimulating and challenging. Consequently, they may experience lower levels of arousal and a sense of disengagement, resulting in feelings of boredom.

Nonetheless, according to the MAC model, individuals exhibit varying capacities to cope with and alleviate boredom. Mathematically talented students, and likely gifted and talented students in general, leveraging their intellectual capabilities and resourcefulness, often excel in finding strategies to combat boredom. They proactively pursue intellectually stimulating and challenging activities, engage in independent exploration and learning, and participate in extracurricular pursuits aligned with their interests and passions (Olszewski-Kubilius, 2015). By employing these strategies, they effectively restore a sense of arousal and meaningful engagement, thereby mitigating their experience of boredom (Westgate and Wilson, 2018).

The MAC model offers a potential explanation for the variation in boredom tendencies among mathematically talented adolescents. It suggests that these individuals may discontinue activities prematurely when they perceive them as lacking in challenge, which aligns with their propensity to experience boredom easily. The dislike of repetition and routine is a common sentiment among gifted individuals in the literature (Steinberger et al., 2017; Hinterplattner et al., 2022). However, their proactive approach to finding stimulating activities and their ability to engage in independent pursuits contribute to their ability to alleviate and overcome boredom.

For RQ2. “Is boredom related to school attitudes and psychological well-being in this population?,” the lack of novelty and challenge in educational settings can manifest through repetitive routines, limited choices, and a lack of excitement. These components align with previous literature that has identified them as potential sources of boredom among gifted individuals (Kerr et al., 1988; Kaplan and Silverberg, 1991; Gallagher and Gallagher, 1995).

The correlation between a lack of interest and negative attitudes toward school and teachers is significant in mathematically talented students, and can also impact students’ future careers and personal development. There are several potential reasons why a lack of interest might lead to negative attitudes and lower motivation. One possibility is that students who are not excited about learning may find it difficult to see the relevance of school to their lives. Another possible explanation is that school is often seen as a structured and rigid environment, which can be displeasing for gifted and talented students in general (Steinberger et al., 2017; Hinterplattner et al., 2022).

Interpersonal relationships are likely to be more strongly related to the affective and social aspects of boredom as noticed in the interviews, rather than to the cognitive or academic aspects (Goetz et al., 2008; Pekrun et al., 2009). External factors, such as home life or social issues, were not examined in this study but were mentioned briefly by some mathematically talented students; these factors can also impact student interest in school and overall motivation.

For RQ3. “What factors impact their perception of boredom?,” we found that mathematically talented students tend to have a simpler time initiating their interests and enthusiasm and can derive meaning from a variety of activities. Encouraging students to discover constructive ways to deal with boredom can enable them to actively pursue engaging and challenging activities that foster personal growth and the development of their skills and interests (Moynihan et al., 2017; Zhang et al., 2017).

Extracurricular activities can offer a break from the academic routine and provide a sense of balance and fulfillment (Craft, 2012). Independent learning experiences provides gifted and talented students in general with a sense of autonomy and intellectual freedom that can help them stay motivated and engaged in their learning, even when they find the regular curriculum boring or unchallenging (Maker, 1986). They may be more likely to be self-directed learners who are less reliant on external motivators like teacher approval or grades.

Peer relationships can take many forms; including participating in extracurricular leisure activities (e.g., skating, golfing, video gaming, etc.), attending specialized programs or events, and forming school groups. Furthermore, these types of groups can offer opportunities for healthy collaboration and personal growth, which are important for the development of gifted and talented students in general (Subotnik et al., 2011), e.g., “*Being with my friends, the boredom is gone*.” In addition, gifted students may encounter challenges in finding like-minded peers who share their interests and abilities. Thus, it becomes crucial for their social and emotional well-being to spend time with peers whom they feel a sense of comfort, even if the conversations do not necessarily revolve around academic subjects (Shah and Mehta, 2021).

Mathematically talented students from rural areas have encountered different educational experiences prior to entering the enrichment program, which may result in finding the classes more demanding or fast-paced. Rural students with exceptional talent often face limited access to quality education, characterized by lower-quality schools, scarce resources, and fewer qualified teachers (Kettler and Jones, 2008). Consequently, gifted students in rural areas may struggle to receive the advanced instruction and enrichment opportunities necessary to match the academic level of their urban counterparts. The loss of in-person social interactions due to COVID-19 may have impacted students’ sense of community and belonging at school (Shah and Mehta, 2021). Furthermore, their socioeconomic status, often characterized by low-income households, can further constrain their access to resources and extracurricular opportunities, such as books and technology, that facilitate academic growth to reach their full advanced potential (Kettler and Jones, 2008).

### Limitations and implications for the future

First, our sample size was small, which means we should be cautious when generalizing our findings to larger populations of talented students. A point to consider is that the study sample is likely not representative of the broader population of adolescents, as they comprise a particular subset. However, the use of a niche sample such as mathematically talented students participating in advanced enrichment was a deliberate characteristic of the study; this allowed for a more in-depth exploration of the experiences of this specific group.

Moreover, while the scale used to measure boredom in this study has been previously validated in Mexico, no information on additional validation studies in other Latin American countries. The accuracy of any psychometric instrument can affect validity of the results. Therefore, it would be useful for future studies to explore alternative measures of boredom.

Other scales measuring boredom can be explored, as accuracy of scales is a fundamental point when conducting psychological studies relying on psychometric instruments. To ensure the robustness and applicability of findings, studies comparing multiple measures of boredom can be useful; particularly in the light of our qualitative results, where we could detect a philosophical confusion in the concept of boredom.

## Conclusion

In summary, mathematically talented students typically experience less difficulty when it comes to initiating their interests and enthusiasm, and they can find meaning in various activities with relative ease. However, they tend to become bored faster than their peers. Boredom in this population is multifaceted, as it includes the absence of meaning, superficial entertainment, and time wasted in non-meaningful activities. Factors that contribute to boredom include waiting for others who learn at a slower pace, limited choices, lack of novelty, absence of intellectual challenge, and the influence of teachers and social dynamics. Moreover, mathematically talented students reported different strategies to cope with boredom, such as engaging in inner intellectual stimulation, seeking entertainment and escape, pursuing independent learning opportunities, participating in organized extracurricular activities, seeking the company of like-minded peers, and engaging in extreme sensation-seeking behavior. Constructive ways of combating boredom can help them navigate underchallenging environments and actively pursue activities that contribute to their talent development and personal growth.

## Data availability statement

The raw data supporting the conclusions of this article will be made available by the authors, without undue reservation.

## Ethics statement

Ethical approval was not required for the study involving human samples in accordance with the local legislation and institutional requirements. Written informed consent for participation in this study was provided by the participants’ legal guardians/next of kin.

## Author contributions

AV conceptualized and designed the study methodology, reviewed the literature, directed quantitative and qualitative data collection, conducted quantitative and qualitative analyses, drafted the initial manuscript, and edited the manuscript until its final version. MM reviewed the literature, coordinated qualitative data collection and trained focus groups leaders, conducted qualitative analyses, and drafted several iterations of the manuscript. LB served as liaison for participant recruitment and operational parts of the study, participated in qualitative analyses, and provided notable feedback for writing. AV, MM, and LB contributed to manuscript revision, read, and approved the submitted version. All authors contributed to the article and approved the submitted version.
